# Venetoclax in the Treatment of Chronic Lymphocytic Leukemia: Evidence, Expectations, and Future Prospects

**DOI:** 10.7759/cureus.8908

**Published:** 2020-06-29

**Authors:** Saba Tariq, Sundus Tariq, Maliha Khan, Aysha Azhar, Mukhtiar Baig

**Affiliations:** 1 Pharmacology and Therapeutics, The University of Faisalabad, Faisalabad, PAK; 2 Physiology, The University of Faisalabad, Faisalabad, PAK; 3 Oncology, The University of Texas MD Anderson Cancer Center, Houston, USA; 4 Post Graduate Research Department, Madina Teaching University, Faisalabad, PAK; 5 Clinical Biochemistry, King Abdulaziz University, Jeddah, SAU

**Keywords:** venetoclax, chronic lymphocytic leukemia, pharmacokinetics, pharmacodynamics

## Abstract

Chronic lymphocytic leukemia (CLL) is the most common form of leukemia in the western adult population; it is also prevalent worldwide. The B cell lymphoma-2 (BCL-2) family proteins play a key role in regulating intrinsic apoptosis and, in many cancers, are the main culprits behind tumor survival and therapy resistance. Hence, the role of BCL-2 inhibitors is very beneficial in the treatment of CLL. Venetoclax is the first selective, orally bioavailable BCL-2 inhibitor.

This review article discusses factors such as the pharmacokinetics, pharmacodynamics, acquired resistance to venetoclax, responders vs. non-responders in venetoclax monotherapy, and the synergistic role of venetoclax with other drugs in detail. Venetoclax is the first BH3 mimetic drug and selective BCL-2 inhibitor that has received FDA approval. This drug has proved to provide good therapeutic responses in CLL patients irrespective of the presence of adverse clinical or genetic features, including in patients with relapsed or refractory forms of CLL. We anticipate that novel combination therapies, including venetoclax and immunotherapy, will further alter the treatment landscape for patients with relapsed CLL, particularly for those with deletion 17p (del 17p) CLL, which carries a very poor prognosis.

## Introduction and background

Chronic lymphocytic leukemia

Chronic lymphocytic leukemia (CLL) is a hematological monoclonal neoplastic disorder. It is the most common form of leukemia in the western adult population [1], accounting for approximately 30% of all leukemias in this group [2]. It is characterized by the proliferation of incompetent, poorly formed, and dysfunctional CD5/CD23-B lymphocytes, thereby leading to their accumulation in the peripheral blood, lymphoid tissues, and bone marrow, result­ing in lymphocytosis, lymphadenopathy, splenomegaly, and leukemia cell infiltration of the marrow [3].

CLL represents 22-30% of all leukemias worldwide, with an incidence between <1 and 5.5 per 100,000 people per year. According to a study carried out in 2004, the countries with the highest incidence rates were Australia, the United States (US), Ireland, and Italy [4]. In the US, the annual incidence of CLL is nearly 4.6 cases per 100,000 persons/year, with 4,500 deaths and >15,000 newly diagnosed cases reported per year. In 2015, there were approximately 14,620 new CLL cases reported in the US alone [5]. More than 95% of patients were older than 50 years, with a median age at diagnosis of 71 years [2]. The occurrence is slightly more common in males than in females of the same age group [3] and is less frequent in individuals with Asian and Middle Eastern ancestry [6].

Treatment strategy

Multiple factors must be taken into consideration before treatment selection. These include the patient’s condition at diagnosis, clinical stage of the disease, response to previous chemotherapy, and the molecular and cytogenetic makeup of the patient. For example, in those with deletion 17p (del 17p) CLL, options that lead to therapeutic responses are fewer; hence, selecting a suitable treatment option for this population is critical [7]. A wide variety of treatment options are available for patients with CLL. These include chemotherapy, a combination of chemotherapy and immunotherapy, and drugs that target the signaling pathways that facilitate the growth and survival of CLL cells [e.g., B cell antigen receptor (BCR) signaling and B-cell lymphoma-2 (BCL-2)] [8]. Currently, the first-line treatment for patients in good condition and without significant comorbidities is predominately chemotherapy (chlorambucil, fludarabine, cyclophosphamide, and bendamustine) and combination therapy with monoclonal antibodies to CD20 (rituximab). According to the CLL 3 Trial, the fludarabine plus cyclophosphamide (FC) and cladribine plus cyclophosphamide (CC) regimens have similar therapeutic efficacy as the first-line treatment of CLL [9].

Proto-oncogenes, particularly BCL-2 genes, are mainly responsible for the resistance to programmed cell death seen in patients with CLL [10]. It is interesting to note that BCL-2 expression is increased in 95% of the patients with CLL [11]. One of the important factors that result in overexpression of BCL-2 is the hypomethylation of the BCL-2 gene, which leads to an increase in the activity, as indicated by histone H3 lysine 27 (H3K27) acetylation chromatin analysis. The absence of micro RNA due to post-transcriptional regulation and an increase in the expression of myeloid cell leukemia 1 (MCL1) protein maybe the other mechanisms of resistance [12,13]. BCL-2 overexpression results in the formation of aberrant signaling pathways, which lead to the proliferation and survival of BCL-2 cells. This has been explained by a transgenic mouse model of t(14;18) translocation where the overexpression of BCL-2 led to the activation of the nuclear factor-kB (NFkB) pathway or the overexpression of tumor necrosis factor (TNF)-receptor-associated factor2 (TRAF2) led to the activation of NF-kB and c-Jun N-terminal kinase (JNK). It was found that only those mice with both t(14;18) and TRAF2 overexpression developed an aggressive form of CLL [14]. This model closely mimics CLL, in which NF-kB is also inherently activated [15].

Venetoclax

**Figure 1:**
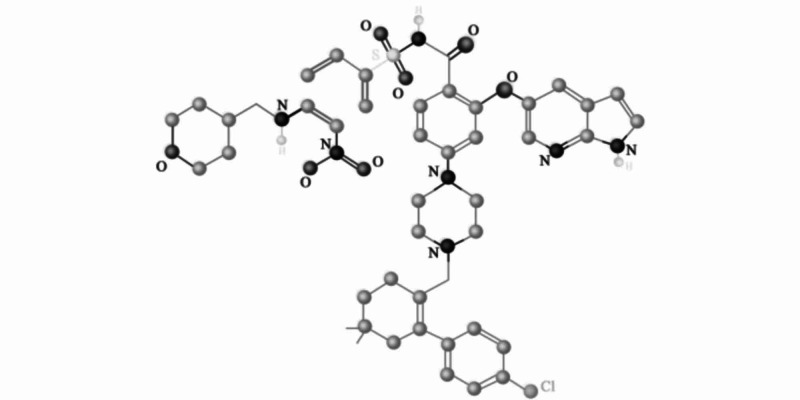
Structure of venetoclax

BCL-2 family proteins play a crucial role in regulating intrinsic apoptosis and are the main culprits behind tumor survival and therapy resistance in many cancers [16,17]. Therefore, the role of BCL-2 inhibitors is very beneficial in the treatment of CLL [18-21]. Venetoclax is the first selective, orally bioavailable BCL-2 inhibitor. The molecular weight for venetoclax is 868 Da. The main advantage of venetoclax over other agents is that it has a high binding affinity for BCL-2 receptors and it very selectively inhibits BCL-2, maintaining anti-apoptotic activity in cancer cells (Figure 1)[22]. Monotherapy with this agent facilitates a rapid reduction in the disease burden with a high overall response of about 80% and a complete response of 6-20% in patients with relapsed or refractory CCL, including those with chromosome 17p deletions [18,20].

## Review

Pharmacokinetics

The study of chemotherapeutic drugs on healthy volunteers has always been a daunting task. However, phase I and II trials have provided useful insights into the pharmacology of venetoclax. The half-life of venetoclax after administration of a single dose of 50 mg is about 16-19 hours, and it is highly plasma protein-bound (>99%) [18,23,24]. Clinical trials have shown that a steady-state is typically achieved within a week with daily dosing, associated with minor, non-problematic accumulation in the body [23,24]. In a study conducted on 505 subjects, the volumes of distributions at steady-state were calculated at 321 (95% CI: 288-340) and 256 (95% CI: 228-276) in the male and female cancer patients, respectively. This indicates its large volume of distribution and extensive tissue binding capacity; and It is predominately metabolized by CYP3A. Apparently, total clearance of the drug from plasma after oral administration (Cl/F) decreases to 19% in the case of moderate CYP3A inhibitors, and it can further decrease to 84% in the case of potent CYP3A inhibitors [25].

The bioavailability of venetoclax is dependent on food and dosage [26]. Peak concentrations are achieved after four to five hours of administration in fasting patients and delayed by approximately two hours when taken with a meal. The maximum concentration (Cmax) and area under the curve (AUC) are also raised by three to five folds when taken with food, especially a high-fat meal [27]. The probable explanation for this effect is that the lipid content in food increases the intestinal lymphatic transport of the drug [28]. This increases the fraction of drug absorbed and bypasses the hepatic first-pass effect, thus increasing the amount of drug reaching the systemic circulation. Therefore, it is recommended that venetoclax be administered once daily with a meal, preferably one rich in fat, as it increases the bioavailability by 4.25 fold [27]. Acid-reducing agents such as omeprazole and cimetidine do not decrease the bioavailability of venetoclax, and factors such as body weight, age, and race affect its pharmacokinetics [24,25]. No dose adjustment is required in mild to moderate hepatic or renal impairment [29]; however, the clearance in severe abnormalities of kidney or liver function has not yet been studied. There is minimal urinary excretion of intact venetoclax [23].

Dosage

Dosage of the drug is administered according to a weekly ramp-up schedule over five weeks to the recommended daily dose of 400 mg. The five-week ramp-up dosing schedule is designed to reduce tumor burden. In this ramp-up schedule, the dose is started at 20 mg in week one and is increased to 50, 100, 200, and 400 mg in weeks two, three, four, and five, respectively [30].

Pharmacodynamics

**Figure 2:**
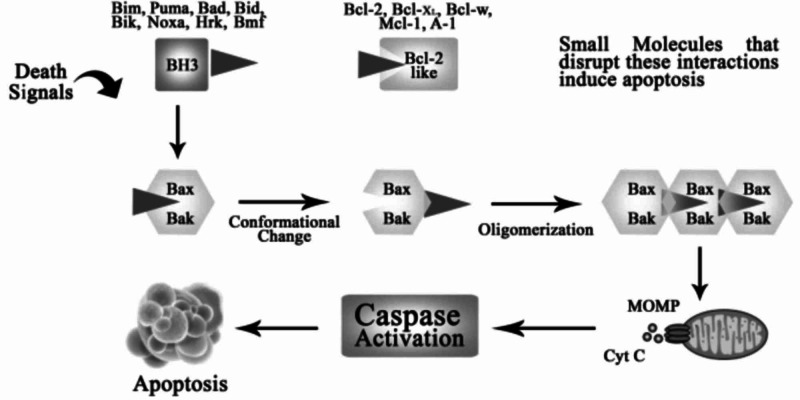
Regulation of apoptosis by BCL-2 family MOMP: mitochondrial outer membrane permeabilization; BCL-2: B-cell lymphoma-2; Cyt C: cytochrome c

Programmed cell death is regulated by the BCL-2 family of proteins. In healthy B-lymphocytes, unnecessary apoptosis is prevented by keeping the death mediators, BAX and BAK, in strict check by BCL-2 and other pro-survival/anti-apoptotic proteins [BCL-2L1 (BCL-XL) or MCL1] (Figure 2) [31-33].

However, when the cells are no longer required, they undergo significant irreversible damage, and apoptosis is initiated by activation of BH3- only proteins (BIM, PUMA), which are naturally occurring antagonists of pro-survival BCL-2 proteins. These pro-apoptotic proteins bind and inactivate BCL-2 and its related pro-survival proteins [34], rendering BAX and BAK free to cause mitochondrial damage and activate intrinsic pathways of apoptosis. In the case of CLL, the overexpression BCL-2 leads to inappropriate cell survival [35], tumor formation [36], and diminished sensitivity to chemotherapy [37]. Since the antagonistic role of BH3-only proteins towards the BCL-2 is a pivotal step for initiating apoptosis, agents that potently mimic this action were developed to pharmacologically inhibit the pro-survival proteins and initiate programmed cell death in CLL [38].

Initially, a drug named navitoclax was designed. This drug could inhibit BCL-2 receptors as well as B-cell lymphoma-extra large (BCL-XL). BCL-XL also possesses anti-apoptotic activity and is encoded by the BCL-2 genes. In early clinical trials, the dose of navitoclax had to be limited because of the potential side effects of thrombocytopenia. Later, it was discovered that the thrombocytopenia was secondary to the navitoclax- mediated inhibition BCL-XL and was independent of the dose. This necessitated the need for a more specific agent that could selectively inhibit BCL-2 without affecting BCL-XL. Later on, venetoclax was designed to mediate apoptosis in cells with overexpression of BCL-2 without the effects of thrombocytopenia [39]. A significant number of CLL patients show increased responsiveness when treated with chemotherapy, the mechanism of action for which is the induction of apoptosis through tumor protein p53 (TP53). Clonal evolution can lead to mutations in as much as half of the patients in a relapsed or refractory disease setting.

A mutation in TP53 or deletion of parts of chromosome 17 can impair the TP53 function. As a result, the cells' ability to sense DNA damage caused by cytotoxic agents, and the initiation of an appropriate apoptotic response greatly decreases [40]. In an in vitro study, venetoclax was found to kill CLL cells, murine lymph node B cells, and RS4;11 human lymphoblast cell lines irrespective of TP53 deletion, mutation, or function. The study further demonstrated that TP53 status does not affect clinical parameters of response to venetoclax [41]. Therefore, as per in vitro and in vivo studies, venetoclax acts independently of TP53 to rapidly induce apoptosis of CLL.

Acquired resistance to venetoclax

Resistance is one of the major problems in cancer chemotherapy. There are many possible hypotheses for the resistance of venetoclax. One of the most likely mechanisms of resistance is the up-regulation of other proteins members of the BCL-2 anti-apoptotic family. These members include BCL-2A1, BCL-XL, BCL-W, and MCL [42]. Stimulation with CD40 and interleukin-4 (IL-4) causes overexpression of BCL-XL in CLL patients. This can lead to resistance to much higher doses of venetoclax [42]. Another hypothesis has stated that resistance is mainly because of proliferation centers that send kinase-mediated survival signals. Another factor in the resistance to venetoclax is individual variation in these signals. These signals may up-regulate anti-apoptotic proteins, as described earlier [43]. One of the important strategies to overcome resistance is to switch between different therapies. Patients with venetoclax resistance show good responses when treated with dasatinib and ibrutinib [42]. Sunitinib is a small molecule, orally available and approved by the FDA for renal cell carcinoma. Literature shows that sunitinib inhibits multiple tyrosine kinase receptors and can be used to overcome venetoclax resistance in CLL patients. It has been found to be more effective than dasatinib and ibrutinib in overcoming resistance to this drug [44]. Nevertheless, it seems the venetoclax resistance might be challenging to overcome with any agent over time. Therefore, it is essential to continue designing and discovering such novel agents that contribute to improvements in chemotherapy.

Responders vs. non-responders in venetoclax monotherapy

Very few clinical trials have been undertaken with venetoclax alone or in combination with other drugs, and they have shown reasonable response rates. Roberts et al. (2016) performed an open-label, multicenter, phase I/II, monotherapy clinical trial in patients with relapsed or refractory CLL, small lymphocytic lymphoma (SLL), or non- Hodgkin's lymphoma. The study was divided into two phases. In the dose-escalation phase, 56 patients received active treatment in one of eight dose groups that ranged from 150 to 1,200 mg of venetoclax per day, out of which three developed tumor lysis syndrome (TLS), leading to one death. Sixty additional patients were treated with a weekly stepwise ramp-up dose in an expansion cohort, increasing from 20, 50, 100, and 200 mg to the final recommended dose of 400 mg per day. No TLS was seen in any patients in this phase. Other main adverse events were diarrhea (52%), upper respiratory tract infection (URTI) (56%), nausea (55%), neutropenia (52%), fatigue (46%), and cough (35%). The overall response rate (ORR) with venetoclax in all patients in the escalation cohort and expansion cohort was 79%, 77%, and 82%, respectively, while the complete response rate (CRR) was 20%, 30%, and 10%, respectively. The ORR was better in patients of <70 years of age (83%) than with patients of ≥70 years in age (71%). Patients previously resistant to fludarabine showed an ORR of 79% and CRR of 16%. Patients who were not resistant to fludarabine showed better ORR and CRR of 82% and 27%, respectively. Among all the patients who had a response, the estimated durability of response was 75% (95% CI: 64-84) at 15 months. The overall survival estimate during the two-year duration for all the patients was 84% [18].

Another multicenter, monotherapy, phase 2, single-arm clinical trial with venetoclax in patients with relapsed or refractory del(17p) CLL, was carried out by Stilgenbauer et al. (2016). In this trial, 107 patients were put in the main cohort and 50 in the safety expansion cohort to evaluate the safety and updated tumor lysis prophylaxis and management measures. Patients were administered venetoclax once daily with a weekly ramp-up schedule of four to five weeks (20, 50, 100, 200, and 400 mg) followed by 400 mg per day, which was continuous dosing until disease progression or discontinuation for another reason. The most common grade 3-4 adverse events were neutropenia (40%), infection (20%), anemia (18%), and thrombocytopenia (15%). Serious adverse events occurred in 59 (55%) patients, irrespective of their relationship to treatment, with the most common (≥5% of patients) being pyrexia and autoimmune hemolytic anemia (7%), pneumonia (6%), and febrile neutropenia (5%). Eleven patients died in the study within 30 days of the last dose of venetoclax: seven due to disease progression and four from an adverse event (none assessed as treatment-related). The ORR was 85%, indicating that this monotherapy can be tolerated well and is active in relapsed or refractory CLL patients [20].

Jones et al. (2015) performed a phase II, double arm, venetoclax monotherapy clinical trial in 28 patients with relapsed or refractory CLL. Twenty-two patients previously treated with ibrutinib for a median duration of 15.5 months entered in arm A, and six patients previously treated with idelalisib entered in arm B for a median duration of 9.7 months. Patients were administered venetoclax once daily (20, 50, 100, 200, and 400 mg) over five weeks with a weekly ramp-up plan. Adverse events seen in more than 25% of treatment subjects were neutropenia (57%), anemia (35%), nausea (32%), and diarrhea (32%). The grade 3-4 adverse events seen in more than 10% of treatment subjects were neutropenia (43%), anemia (29%), and thrombocytopenia (18%). The ORR was 53% [45] (Table 1).

**Table 1:** Responders in venetoclax monotherapy in CLL patients CLL: chronic lymphocytic leukemia; SLL: small lymphocytic lymphoma; IGHV: immunoglobulin heavy-chain variable region; TP53: tumor protein p53; ORR: overall response rate; TLS: tumor lysis syndrome

Study	Study design	Patients	Interventions	Results			Toxic effects
				Responders	Complete remission	PFS	
Robert et al. (2016) [18]	Phase I/II; phase I dose-escalation study (n=56); after adjustment continued as phase II expansion cohort (n=60)	N=116; relapsed or refractory CLL (88%), SLL (12%), deletion 17p CLL (30%), deletion 11q CLL (27%), IGHV-mutated (17%), previous fludarabine-treated (86%), fludarabine-resistant (60%)	One of eight dose groups, 50 to 1,200 mg/day oral; weekly stepwise ramp-up in doses as high as 400 mg per day	ORR (79%), phase 1 (77%), phase 2 (82%), fludarabine-resistant (79%), deletion 17p CLL (71%), deletion 11q CLL (82%), IGHV-mutated (94%)	Complete response rate (20%), phase 1 (30%), phase 2 (10%), fludarabine-resistant (16%), deletion 17p CLL (16%), deletion 11q CLL (11%), IGHV-mutated (29%)	15-month progression-free survival estimate for the 400-mg dose groups was 69%	Clinical TLS in three of 56 patients with one death, not seen after dose escalation adjustment. Mild diarrhea (52%), upper respiratory tract infection (48%), nausea (47%), grade 3-4 neutropenia (41%)
Stilgenbauer et al. (2016) [20]	Phase II, single-arm	N=107 (main cohort), relapsed or refractory CLL with deletion 17p; n=50 (safety expansion cohort) continued	Started with weekly ramp-up schedule (20, 50, 100, 200, and 400 mg) once-daily dose for four to five weeks, continued at 400 mg per day until disease progression or discontinuation for another reason	ORR 85%	8%	72% at the 12-month period	Grade 3-4 neutropenia (40%), infection (20%), anaemia (18%), and thrombocytopenia (15%)
Jones et al. (2015) [45]	Phase II, double-arm	N=28; relapsed or refractory CLL post-ibrutinib (arm A) or idelalisib (arm B); deletion 17p CLL (41%), deletion 11q CLL (55%), TP53-mutated (36%)	Started with weekly ramp-up schedule (20, 50, 100, 200, and 400 mg) dose once daily	53%	0	-	Adverse events in >25% of patients were neutropenia (57%), anemia (35%), and diarrhea (32%); grade 3-4 in 10% of patients were neutropenia (43%), anemia (29%), and andthrombocytopenia (18%)

Synergistic role of venetoclax with other drugs

The treatment protocols of relapsed or refractory CLL are changing due to the development of resistance to monotherapy and also with a view to improving the patient outcome of this disease as it continues to have a relatively poor prognosis despite the wide variety of treatment options available. Venetoclax monotherapy, as shown in the previous trials, is active and well-tolerated; but unfortunately, experimental evidence has shown mutations in the BCL-2, BH3 domain, and C-terminal transmembrane domain of BAX on continuous exposure to venetoclax, thereby leading to ultimate resistance [46]. The synergistic role of venetoclax has also been observed in combination with other drugs in various clinical trials. A phase Ib trial of venetoclax with bendamustine/rituximab (VEN+BR) or bendamustine/obinutuzumab (VEN+BG) was conducted by Stilgenbauer et al. (2016) in patients with relapsed/refractory (R/R) (n=30) or previously untreated CLL (1L) (n=25). The treatment was started with six months of combination therapy in two schedules: A and B. In schedule A, venetoclax was administered before BR or BG and afterward in schedule B. Venetoclax 100 to 400 mg/day in a gradual ramp-up fashion was given with BR/BG in three+three dose-escalation cohort, followed by a safety expansion cohort of 400 mg, continued as a single-agent VEN until unacceptable toxicity, disease advancement, or up to one-year total VEN [47]. The adverse events seen in >20% of the 47 patients who received VEN+BR (30 R/R; 12 on schedule A at doses 100-400 mg, 18 on schedule B at 400 mg) include neutropenia (63%), thrombocytopenia (47%), any infections and infestations (73%), anemia (43%), and diarrhea (43%). Grade 3-4 adverse events were seen in >10% of the patients and included neutropenia (63%), thrombocytopenia (27%), any infections and infestations (27%), anemia (20%), and diarrhea (10%). The adverse events that were seen in >20% of the 47 patients who received VEN+BR (17 1L, all at 400 mg: six on schedule A, 11 on schedule B) were neutropenia (76%), thrombocytopenia (59%), any infections and infestations (53%), anemia (35%), and diarrhea (29%); grade 3-4 adverse events seen in >10% of the patients included neutropenia (71%), thrombocytopenia (24%), any infections and infestations (0%), anemia (29%), and diarrhea (0%).

The adverse events seen in >20% of the eight patients who received VEN+BG (all on schedule B at 400 mg) were neutropenia (25%), thrombocytopenia (63%), any infections and infestations (63%), anemia (0%), and diarrhea (38%). In comparison, grade 3-4 adverse events seen in >10% of the patients were neutropenia (25%) and thrombocytopenia (63%). Venetoclax was discontinued early due to toxicity (mainly due to neutropenia and thrombocytopenia) in seven patients on VEN+BR (R/R), two patients on VEN+BR (1L), and one patient on VEN+BG. Combination drugs, bendamustine, rituximab, and/or obinutuzumab were discontinued in 10 patients with VEN+BR (R/R), four patients on VEN+BR (1L), and two patients on VEN+BG. A median of four cycles of B was completed even with early discontinuation. The response was seen in all evaluated patients, with complete remission (CR) in more than half of patients with manageable toxicities and no TLS [47].

Fischer et al. (2016) evaluated the safety and efficacy of venetoclax and obinutuzumab (G) in comparison with chlorambucil (C) and obinutuzumab in patients with IL in an open-label, multicenter, run-in phase of a randomized trial. Venetoclax was administered orally in a ramp-up dose fashion (20, 50, 100, 200, up to 400 mg) starting on the 22nd day of the first cycle. Obinutuzumab (G) was given IV starting with 100, 900, 1,000, and 1,000 mg on days one, two, eight, and 15, respectively, followed by 1,000 mg on day one for cycles two to six. Initially, six cycles of venetoclax plus obinutuzumab were given, followed by six cycles of venetoclax monotherapy. The most common adverse events observed were infusion-related reactions (75%), neutropenia (67%), infections (67%), pruritus (58%), and diarrhea (50%). Grade 3-4 adverse events included neutropenia (58%), infections (17%), and thrombocytopenia (17%). Laboratory TLS was seen in 17% of the patients with no evidence of clinical TLS. The ORR was 100%, with CR in 58% of the patients and progression-free survival (PFS) in 100%, evaluated at the end of 15 months, indicating that the method was effective and well-tolerated; and this trial has continued [48].

Flinn et al. conducted another phase 1b trial (2015) evaluating the efficacy of venetoclax and obinutuzumab combination therapy in R/R or 1L patients with CLL. Treatment was started with either schedule A (VEN) or schedule B (G) with a three+three design and 100-600 mg/day of venetoclax cohorts, given in gradual ramp-up fashion. Combination therapy continued for six cycles, followed by venetoclax monotherapy in R/R patients until disease progression and an additional six months of venetoclax monotherapy in 1L patients. Infectious adverse events and diarrhea were seen in 50% of patients, while infusion-related reactions and neutropenia was seen in 40% and 37% of the patients, respectively. Grade 3 and grade 4 neutropenia was seen in 34%, and 12% of the patients, respectively, and laboratory TLS was observed in 12% with no clinical TLS seen. One death in cohort 1, with R/R CLL, occurred secondary to acute respiratory failure. The ORR, evaluated in 17 patients, was found to be 100% and CR/complete remission with incomplete hematologic response (CRi) was seen in 23.5% of the patients. This again confirmed the safety and tolerability of VEN+G in both R/R and 1L patients [49].

A phase Ib clinical trial was undertaken on 49 patients with relapsed or refractory CLL or SLL. Venetoclax was used in combination with rituximab. Venetoclax was started with a once-daily dose of 20 or 50 mg, increased in a ramp-up fashion to final-cohort doses of 200-600 mg/day in five dose-escalation cohorts (n=41), while 400 mg/day in safety-expansion cohort (n=8), tailed by rituximab, for a total of six doses given every four weeks. Of note, 92% and 59% of patients had received prior rituximab and fludarabine therapies, respectively. Patients having rituximab-refractory (R-ref) and fludarabine-refractory (F-ref) disease were 29% and 18%, respectively. Twelve patients refused to continue the study: six because of PD (five were Richter's transformation), three due to adverse events [neuropathy, TLS, and myelodysplasia (heavily pretreated and hypocellular marrow at study entry; patient achieved minimal residual disease (MRD)-negative complete remission (CR) with incomplete marrow recovery (CRi) and proceeded to transplant)]. Three withdrew consent (one after achieving MRD-negative CR). The most common adverse events seen in more than 25% of treatment subjects were neutropenia (55%), diarrhea (53%), nausea (49%), and upper respiratory infection (45%). Grade 3-4 adverse events seen in more than 10% of treatment subjects were neutropenia (53%), thrombocytopenia (16%), anemia (14%), febrile neutropenia (12%), and leukopenia (10%). One death occurred due to TLS, after which the protocol was modified. ORR was 86% in all treatment subjects, 89% in del (17p), 56% in F-ref, 84% in IGHV-unmutated. The PFS measured at 12 months' duration for all patients, del (17p), F-ref, and IGHV-unmutated was 87%, 89%, 56%, and 83%, respectively, while PFS at 24 months was 84%, 78%, 56%, and 83%, respectively. The overall survival rate at 12 months was 94%, 89%, 89%, and 89%, respectively, indicating that this combination therapy is active and highly responsive with a tolerable safety profile (Table 2) [50].

**Table 2:** Responders in venetoclax combination therapy in CLL patients CLL: chronic lymphocytic leukemia; SLL: small lymphocytic lymphoma; IGHV: immunoglobulin heavy-chain variable region; TP53: tumor protein p53; ORR: overall response rate; TLS: tumor lysis syndrome; VEN: venetoclax; B: bendamustine; R: rituximab; G: obinutuzumab; PFS: progression-free survival; R-ref: rituximab-refractory; F-ref: fludarabine-refractory; MRD: minimal residual disease

Study	Study design	Patients	Interventions	Results			Toxic effects
				Responders	Complete remission	PFS	
Stilgenbauer et al. (2016) [47]	Venetoclax with bendamustine/rituximab (VEN+BR) or bendamustine/obinutuzumab (VEN+BG); phase Ib (recruiting)	Relapsed or refractory CLL (R/R), previously untreated CLL (1L); VEN+BR (R/R, n=30), evaluated n=24, del 17p (25%), del 11q (29%), del 13q (54%); VEN+BR (1L, n=17), evaluated n=15, Del 17p (13%), del 13q (67%); VEN+BG (1L, n=8), evaluated n=5, del 17p (20%), del 11q (20%), del 13q (80%)	Schedule A = VEN before BR/BG; schedule B = VEN after BR or BG; 47 patients received VEN+BR: 30 R/R (12 on schedule A at doses 100-400 mg, 18 on schedule b, all at 400 mg), and 17 1L (all at 400 mg: six on schedule A, 11 on schedule B). Eight patients received VEN+BG: all on schedule B at 400 mg	VEN+BR (R/R) 96%, VEN+BR (1L) 100%, VEN+BG (1L) 100%	VEN+BR (R/R) 20%, VEN+BR (1L) 43%, VEN+BG (1L) 43%		Severe adverse events in 21 patients including erythema, febrile neutropenia, vomiting, nausea, and infection (43% R/R VEN+BR, 35% 1L VEN+BR, 25% 1L VEN+BG)
Fischer et al. (2016) [48]	Venetoclax plus obinutuzumab (VEN+G); open-label, multicenter, run-in phase of phase III randomized trial	Previously untreated CLL (1L) (n=12), cytogenetic (n=8), del 17p (25%), del 11q (25%), TP53-mutated (25%), TP53 deleted (25%), IGHV-mutated (evaluated n=7), (14%)	Six cycles (VEN+G) followed by six cycles (VEN) I/V G (100, 900, 1,000 mg on day one, two, eight, and 15 of cycle one; on day one, 1,000 mg for two to six cycles) VEN oral in ramp-up dose weekly starting at day 22 of cycle one (20, 50, 100, 200, and 400 mg)	100%	58%	100% at 15 months	Neutropenia (67%), infections (67%), diarrhea (50%), infusion-related reaction (75%), pruritus (58%). Grade 3-4 adverse events included neutropenia (58%), infections (17%), and thrombocytopenia (17%)
Flinn et al. (2015) [49]	Venetoclax plus obinutuzumab (VEN+G), phase Ib, phase 3 continued	N=32, relapsed or refractory CLL (R/R) n=26, previously untreated CLL (1L) n=6	Three+three design with 100-600 mg /day venetoclax cohorts	100% among 17 evaluated patients	23.5% among 17 evaluated patients	-	Any infectious adverse event (50%), diarrhea (50%), infusion-related reactions (40%), neutropenia (37%). Grade 3 neutropenia (34%), grade 4 neutropenia (12%)
Ma et al. (2015) [50]	Venetoclax plus rituximab (VEN+R), phase Ib	N=49, relapsed or refractory CLL (48) or SLL (1), R-ref 29%, F-ref 18%, del(17p) 18%, IGHV-unmutated 39%	VEN (per day 20/50 mg) to final cohort doses (200-600 mg per day) tailed by R, total six doses in every four weeks, final 400 mg selected to move forward	ORR 86%, BM MRD-negative 53%, F-ref 56%, deletion 17p CLL (89%), IGHV- unmutated (84%)	CR 41%, BM MRD-negative 75%, F-ref 44%, deletion 17p CLL (33%), IGHV- unmutated (37%)	PFS 87% at 12 months, PFS 84% at 24 months, overall survival (OS) 94% at 12 months	One fatal TLS prior to protocol modification, grade 3/4 adverse events in >10% of pts: neutropenia (53%), thrombocytopenia (16%), anemia (14%), and febrile neutropenia (12%)

## Conclusions

Increased awareness and knowledge of CLL biology has enabled the synthesis of novel therapies that target specific steps of molecular pathways that facilitate tumor cell survival. Venetoclax is the first BH3 mimetic drug and a selective BCL-2 inhibitor that has received FDA approval. The main advantage of venetoclax over other agents is that it has a high binding affinity for BCL-2 receptors and it very selectively inhibits BCL-2, maintaining anti-apoptotic activity in cancer cells. Moreover, monotherapy with this agent facilitates a rapid reduction in the disease burden with an overall good response rate. This drug has proved to provide good therapeutic responses in CLL patients irrespective of the presence of adverse clinical or genetic features, including in patients with relapsed or refractory forms of CLL. We believe that the emergence of novel combination therapies, including venetoclax and immunotherapy, will transform the treatment landscape for patients with relapsed CLL, particularly those with (del 17p) CLL, which carries a very poor prognosis.
